# Bilateral Renal Denervation Ameliorates Isoproterenol-Induced Heart Failure through Downregulation of the Brain Renin-Angiotensin System and Inflammation in Rat

**DOI:** 10.1155/2016/3562634

**Published:** 2016-09-26

**Authors:** Jian-Dong Li, Ai-Yuan Cheng, Yan-Li Huo, Jie Fan, Yu-Ping Zhang, Zhi-Qin Fang, Hong-Sheng Sun, Wei Peng, Jin-Shun Zhang, Hai-Ping Wang, Bao-Jian Xue

**Affiliations:** Department of Physiology and Life Science Research Center, Hebei North University, Diamond South Road 11, Zhangjiakou City, Hebei 075000, China

## Abstract

Heart failure (HF) is characterized by cardiac dysfunction along with autonomic unbalance that is associated with increased renin-angiotensin system (RAS) activity and elevated levels of proinflammatory cytokines (PICs). Renal denervation (RD) has been shown to improve cardiac function in HF, but the protective mechanisms remain unclear. The present study tested the hypothesis that RD ameliorates isoproterenol- (ISO-) induced HF through regulation of brain RAS and PICs. Chronic ISO infusion resulted in remarked decrease in blood pressure (BP) and increase in heart rate and cardiac dysfunction, which was accompanied by increased BP variability and decreased baroreflex sensitivity and HR variability. Most of these adverse effects of ISO on cardiac and autonomic function were reversed by RD. Furthermore, ISO upregulated mRNA and protein expressions of several components of the RAS and PICs in the lamina terminalis and hypothalamic paraventricular nucleus, two forebrain nuclei involved in cardiovascular regulations. RD significantly inhibited the upregulation of these genes. Either intracerebroventricular AT1-R antagonist, irbesartan, or TNF-*α* inhibitor, etanercept, mimicked the beneficial actions of RD in the ISO-induced HF. The results suggest that the RD restores autonomic balance and ameliorates ISO-induced HF and that the downregulated RAS and PICs in the brain contribute to these beneficial effects of RD.

## 1. Introduction

Heart failure (HF) is characterized by cardiac dysfunction along with activation of sympathetic nervous system and reduction of the vagal activity [1]. It has been shown that circulating renin-angiotensin system (RAS) components such as angiotensin (ANG) II and aldosterone and proinflammatory cytokines (PICs) such as tumor necrosis factor-alpha (TNF-*α*), interleukin (IL-) 1*β*, and IL-6 are increased in humans and experimental animals with HF [2–4]. Recently, the link between these peripheral systems activated in HF and the central nervous system (CNS) as a source of neurohumoral drive has been well established. In this context, the lamina terminalis (LT) and hypothalamic paraventricular nucleus (PVN) have emerged as sites that sense humoral signals generated peripherally in response to the stresses of HF and lead to augmented sympathetic drive [2, 5]. Accumulating studies have demonstrated that HF upregulates expression of ANG II type 1 receptor (AT1-R) and PICs in the LT and PVN and that the RAS and PICs synergistically interact in these cardiovascular nuclei to enhance sympathetic activity [6, 7], which further promotes cardiac impairment and leads to deterioration of cardiac function. Conversely, central blockade of AT1-R or TNF-*α* production inhibits sympathetic hyperactivity and improves left ventricular remodeling and dysfunction after myocardial infarction [6, 8, 9].

Renal nerve plays a critical role in the pathogenesis and progression of cardiovascular diseases including hypertension and HF [10]. Several clinical trials have demonstrated that renal denervation (RD) by delivery of radiofrequency pulses from a catheter positioned in the renal artery lowers blood pressure (BP) in patients with resistant hypertension [11] although its effectiveness seems to be questioned based on a recent randomized trial [12]. In different animal models of experimental hypertension, RD prevents the development or slows down the progression of hypertension [10, 13]. Additional beneficial effects of RD also include a reduction in left ventricular hypertrophy and an improvement of diastolic function [11, 14, 15]. The REACH-pilot study has recently shown that, in patients with HF with reduced ejection fraction, BP remained stable after RD, whereas the 6 min walk test improved [16]. Studies in several experimental models of HF also demonstrated the beneficial effects of RD on cardiac function [17–19]. Particularly, unilateral RD improves autonomic balance in conscious rabbits with HF, suggesting that the interaction between renal nerves and the CNS plays an important role in the pathophysiology of HF [20]. However, whether RD, as a therapeutic treatment for HF, exerts beneficial actions via regulation of the forebrain nuclei involved in modulation of cardiovascular functions remains unclear. In addition, patients with HF often exhibit a low BP that limits the possibility of applying evidence-proven drugs (often associated with further BP reductions) to improve the outcomes [21]. Yet there have been few studies that have investigated the time course of BP and heart rate (HR) changes during RD treatment of HF.

Isoproterenol (ISO), a *β*-adrenergic receptor agonist, elicits myocardial impairment, decreased diastolic and systolic function, which is extensively used as an experimental model of HF [22]. Given the synergistic role of central RAS and PICs in producing sympathetic overactivity that deteriorates cardiac functions and progresses into HF [6–9], we hypothesized that RD would downregulate the RAS and PICs in the LT and PVN, thereby rebalancing autonomic activity and ameliorating ISO-induced CHF. To test this hypothesis, we conducted hemodynamic studies including BP and HR recording and evaluation of autonomic and cardiac functions to determine if RD has an influence on BP and HR during ISO administration and if RD restores ISO-induced autonomic imbalance and cardiac dysfunction. To gain further insight into the mechanisms underlying RD beneficial effects, intracerebroventricular (icv) infusion of either AT1-R antagonist irbesartan (Irbe) or TNF-*α* inhibitor etanercept (ENT) during ISO administration was conducted to determine if these treatments could mimic the beneficial actions of RD. Moreover, molecular changes in mRNA and protein expression of the RAS components and PICs in the LT and PVN were characterized to elucidate the interactions between RD and central RAS and PICs.

## 2. Methods

### 2.1. Animals

**Figure 1:**
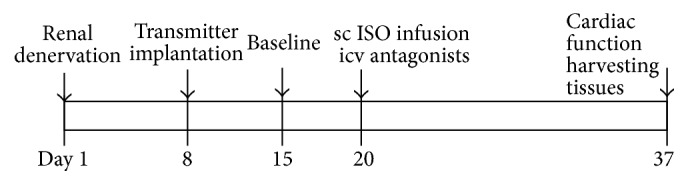
Representative timeline of the study design. One week separated the renal denervation (RD) surgery from the telemetry probe implantation. Experiments were then carried out in peripheral isoproterenol (ISO) treatment with or without central antagonist infusion. Animals were euthanized to collect kidney and brain tissues at the conclusion of the experiments.

Ninety-seven male rats (Wistar, 10–12 wk old) were purchased from Beijing Laboratory Animal Research Center (Beijing, China) and were maintained at an animal facility under barrier-sustained conditions with 12-h light/dark cycle at standard conditions (temperature: 23 ± 2°C, relative humidity: 40%–80%) and free access to standard rat chow and* ad libitum*. All animal procedures were reviewed and approved by the Hebei North University Institutional Animal Care and Use Committee conforming to US National Institutes of Health guidelines. For all surgeries, rats were anesthetized with sodium pentobarbital (1%, 50 mg/kg, IP). For three days following surgery, buprenorphine (0.015 mg, SQ) was given twice per day and the drinking water was supplemented with amoxicillin (1 mg/mL). The timeline for the experimental protocol is shown in Figure 1.

### 2.2. RD and Telemetry Probe Implantation

Rats were subjected to a sham and bilateral RD procedure via a midline approach as previously described [13]. Under surgical microscopy, the renal artery and vein were isolated, and all visible renal nerves from the length of the artery and vein were then carefully removed. The hilus of the kidney was also cleared of fat and any visible nerve fibers followed by painting renal artery and vein with 10% phenol in an alcohol solution to destroy the remaining nerves. After a 7-day recovery period, rats were instrumented with telemetry probes (TA11PA-C40, DSI, St. Paul, MN) through femoral artery for monitoring of BP and HR as previously described [23].

### 2.3. Chronic icv Cannula Implantation and Establishment of ISO-Induced Heart Failure

After 5-day baseline BP and HR recordings, an osmotic pump (model 2ML2, 5 *μ*L/h for 14 days, ALZET) containing ISO (40 mg/kg/d) was implanted subcutaneously in the back for 14 days. Control animals received infusion of vehicle (0.9% saline). In separate group of rats without RD, the icv cannula with an osmotic pump (model 2004, 0.25 *μ*L/h for 4 weeks, ALZET Brain Infusion Kits, ALZET Co.) was implanted into the right lateral ventricle (the coordinates 1.0 mm caudal and 1.5 mm lateral to bregma and 4.5 mm below the skull surface) for chronic infusion of vehicle, AT1-R antagonist Irbe (125 *μ*g/d), or TNF-*α* inhibitor ENT (10 *μ*g/h) for 2 weeks. Thus, the primary study groups (*n* = 6/group) were (1) Sham + saline; (2) RD + saline; (3) Sham + ISO; (4) RD + ISO; (5) icv Irbe + ISO; (6) icv ENT + ISO.

Upon completion of the study, rats were anesthetized and decapitated. Heart, brain, and both kidneys of each rat were harvested, weighed, immediately frozen with liquid nitrogen, and stored at −80°C. Then, the kidneys were assayed for tissue norepinephrine (NE) content; and the brains were analyzed for mRNA expression of the RAS components and PICs as previously described [13, 23]. The microdissected tissue samples for mRNA expression contained the structures lying along the lamina terminalis [LT, i.e., the subfornical organ (SFO), median preoptic nucleus (MnPO), and organum vasculosum of the lamina terminalis (OVLT)] and the paraventricular nucleus of hypothalamus (PVN). Five additional groups (except RD + saline) of rats were given same treatments without BP recording, and their brains were also taken for determining protein expression (*n* = 5/group).

### 2.4. Real-Time RT-PCR Analysis

The total RNA was extracted using RNeasy® Mini Kit (Qiagen, Valencia, CA, USA) and reverse-transcribed into cDNA. mRNA levels for RAS components [angiotensin-converting enzyme (ACE), AT1-R], PICs (TNF-*α*, IL-1*β*, and IL-6), and GAPDH were analyzed with SYRB Green real-time PCR. The sequences for the primers are summarized in Table 1. Real-time RT-PCR was performed with the ABI prism 7300 Sequence Detection System (Applied Biosystems, Carlsbad, CA). The values were corrected by GAPDH and the final concentration of mRNA was calculated using the formula *x* = 2^−ΔΔCt^, where *x* is fold difference relative to control.

### 2.5. Western Blot Analysis

The LT or PVN tissue was homogenized in lysis buffer and the protein concentration in the supernatant was measured with the BCA protein assay Kit (Pierce, Rockford, IL, USA). Equivalent amounts of protein were separated on 12% SDS-polyacrylamide gels and transferred to polyvinylidene difluoride membranes (Millipore Corporation, Bedford, MA, USA). The membranes were blocked with 5% nonfat dry milk and then incubated using primary antibody at 4°C overnight. The primary antibodies used in this study were purchased from Santa Cruz Biotechnology Inc., Santa Cruz, CA, including anti-ACE (sc-20791), anti-TNF-*α* (sc-1350), and anti-*β*-actin (sc-47778). After washing three times, the membranes were incubated with horseradish peroxidase-conjugated second antibody (sc-2004) for 1 h at room temperature. The signal was visualized using the enhanced chemiluminescence (ECL) detection system (Amersham) and the densities of the immunobands were quantitated using NIH ImageJ software (Bethesda, MD, USA). All data were corrected by *β*-actin.

### 2.6. RD Confirmation

Samples of cortical tissue were homogenized and processed to perform an ELISA (GenWay Biotech, San Diego, CA) for NE. To normalize the NE content to protein concentration, a protein assay kit (Pierce, Rockford, IL) was used according to the manufacturer's directions. NE levels of bilateral kidney tissues were compared between the sham groups and RD groups.

### 2.7. Measurement of BP and HR with Telemetry Probes and Cardiovascular Variability and SBRS Analysis

All rats were allowed 7 days to recover from transmitter implantation surgery before any measurements were made. Thereafter, BP and HR were telemetrically recorded and stored with the Dataquest ART® data-acquisition system (version Gold 4.0, DSI, St. Paul, MN). At every three days, BP waveform was continuously recorded for 30 minutes at a sampling rate of 1000 Hz between 10:00 AM and 3:00 PM. These collected data were then used for assessment of cardiovascular variability and spontaneous baroreflex sensitivity (SBRS). This period of day, when rats are relatively inactive, was chosen to minimize the influences of physical activity and arousal.

Beat-to-beat series with mean arterial pressure (MAP) and pulse interval (PI) values were generated by Hemolab (http://haraldstauss.com) and loaded into custom software (Cardioseries version 2.4, http://www.danielpenteado.com) in order to perform cardiovascular variability analysis within time-frequency domains as described previously [24]. The overall variability of MAP and PI was assessed based on the variance of the time series. The variance of PI measurements provided a general measure of HR variability. The root mean square of successive differences (RMSSD) in PI provided a measure of parasympathetic-mediated modulation of HR [25]. In order to better assess autonomic cardiovascular modulation, frequency-domain analysis was also carried out. The variance of MAP measurement provided a measure of BP variability. The spectra were integrated into low-frequency (LF; 0.2–0.75 Hz) and high-frequency (HF; 0.75–3 Hz) bands, and results were expressed in normalized units (nu). To assess the sympathovagal balance, the LF/HF ratio was further calculated. The HF component represents vagal activity and the LF component is more representative of sympathetic activity [24].

SBRS was calculated from spontaneous fluctuations in MAP and HR by using the Hemolab analyzer. The results were expressed as the average slope of the MAP-PI relationships (ms/mmHg).

### 2.8. Evaluation of Cardiac Baroreflexes and Hemodynamic Parameters of Cardiac Function

Six additional groups (*n* = 6–8/group) underwent identical treatment (Sham + saline; RD + saline; Sham + ISO; RD + ISO; icv Irbe + ISO; icv ENT + ISO) were used to evaluate the cardiac baroreflexes and hemodynamic parameters as previously described [26]. Arterial and venous catheters were chronically implanted into the femoral artery and vein for the measurement of BP and administration of drugs, respectively. After three days of recovery, BP was measured with a ML880 BP transducer and continuously recorded using the PowerLab data-acquisition system (chart version 7.2, AD instruments) in conscious rats. Cardiac baroreflexes were evoked by increasing BP with ramp infusions of phenylephrine (PE, 1.0 mg/mL), and by lowering BP with sodium nitroprusside (SNP, 2.0 mg/mL). Baroreflex sensitivity was estimated by calculating the slope of regression lines relating changes in BP and changes in HR during administration of the vasoactive agents.

Under anesthetization, right carotid artery was isolated, through which a cardiac catheter was placed in left ventricle (LV) to record left ventricular pressure (LVP) by using a pressure transducer connected to a PowerLab data-acquisition system (model 16 SP, AD Instruments, Colorado Springs, CO). To assess LV function, left ventricular systolic pressure (LVSP), left ventricular end-diastolic pressure (LVEDP), maximal rate of rise of LVP (+dP/dt_max_), and minimal rate of decrease of LVP (−dP/dt_min_) were measured offline from the LVP data. LVEDP exceeding 15 mmHg was considered as a cut-off value indicative of cardiac failure [27, 28].

### 2.9. Data Analysis

MAP and HR are presented as mean daily values. Differences for MAP and HR were calculated for each animal based on the mean of the 5-day baseline subtracted from the daily MAP or HR during ISO treatment. Two-way ANOVA analysis for the experimental groups was then conducted on daily MAP, HR, or the means of calculated differences. After establishing a significant ANOVA, post hoc analyses were performed with Tukey multiple comparison tests between pairs of mean changes. Same statistical analysis was used to analyze the changes in the parameters of cardiovascular variability, cardiac baroreflex, hemodynamic parameters of cardiac function and differences in NE content, mRNA or protein expression of the RAS components, and PICs in the LT and PVN. All data are expressed as means ± SE. Statistical significance was set at *P* < 0.05.

## 3. Results

### 3.1. Kidney Norepinephrine Content

**Figure 2:**
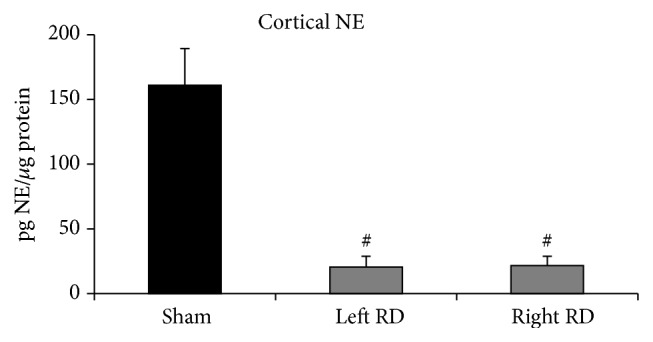
Effect of sham surgery (Sham) and renal denervation (RD) on norepinephrine (NE) content in left and right kidneys (Sham *n* = 17; RD *n* = 29; ^#^ *P* < 0.05 versus Sham).

Bilateral kidneys were collected at the end of the study to determine the extent of denervation 4-5 weeks after procedure. Results of the NE assay clearly showed that RD selectively denervated the kidneys (Figure 2).

### 3.2. Effects of Renal Denervation, Central Blockade of AT1-R, or Inhibition of TNF-*α* on ISO-Induced Cardiac Dysfunction

There were no significant differences in total heart weight (HW) and ratio of HW to body weight (HW/BW) between Sham and RD groups with saline treatment. After ISO treatment, both HW and HW/BW were significantly increased. Either RD or central blockade of AT1-R or TNF-*α* reversed these changes (Table 2).

To evaluate LV function, hemodynamic parameters were measured in anesthetized rats of all the groups. After ISO treatment, rats exhibited signs of cardiac failure. LVSP and the rate of pressure development +dp/dt_max_ and −dp/dt_min_ were significantly reduced while LVEDP was significantly increased (>15 mmHg) in ISO group compared with saline group (*P* < 0.05, Table 2). Either RD, icv Irbe, or icv ENT significantly improved the ISO-induced depression in LVSP, +dp/dt_max_, and −dp/dt_min_, whereas the ISO-induced increase in LVEDP was significantly attenuated (*P* < 0.05, Table 2).

### 3.3. Effects of Renal Denervation, Central Blockade of AT1-R, or Inhibition of TNF-*α* on ISO-Induced BP and HR

**Figure 3:**
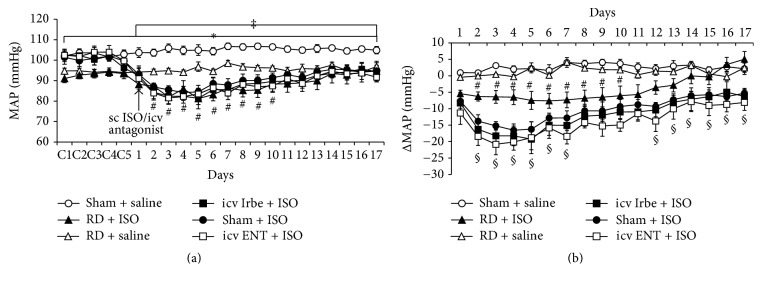
Depressor effects induced by isoproterenol (ISO) in rats with sham surgery (Sham) or renal denervation (RD). This depressor effect was attenuated by bilateral RD, but not by intracerebroventricular (icv) infusion of AT1-R antagonist irbesartan (Irbe) or TNF-*α* inhibitor etanercept (ENT) (a). (b) shows the changes in mean arterial pressure (MAP) after saline or ISO administration in all groups (*n* = 6/group; ^*∗*^ *P* < 0.05 versus RD + saline; ^‡^ *P* < 0.05 versus Sham + ISO; ^#^ *P* < 0.05 versus RD + saline; ^§^ *P* < 0.05 versus RD + ISO).

**Figure 4:**
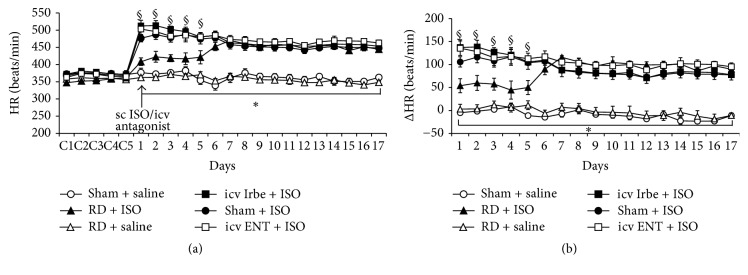
Tachycardiac effects induced by isoproterenol (ISO) in rats with sham surgery (Sham), renal denervation (RD), intracerebroventricular (icv) infusion of AT1-R antagonist irbesartan (Irbe), or TNF-*α* inhibitor etanercept (ENT) (a). (b) shows the changes in heart rate (HR) after saline or ISO administration in all groups (*n* = 6/group; ^*∗*^ *P* < 0.05 versus Sham + ISO or RD + ISO; ^§^ *P* < 0.05 versus RD + ISO).

Baseline MAP and HR were 101.7 ± 2.5 mmHg and 372.3 ± 6.2 beats/min in Sham groups of rats, respectively. RD significantly reduced baseline MAP (93.6 ± 1.9 mmHg, *P* < 0.05) but did not alter baseline HR (356.8 ± 5.9 beats/min, *P* > 0.05). Saline treatment had no effects on these basal BPs and HRs (Figures 3(a) and 4(a)).

In Sham group of rats, ISO resulted in a remarked decrease in MAP throughout treatment period. This decreased MAP was greater in first week and then gradually restored but did not return to baseline level (*P* < 0.05, Figure 3(a)). However, ISO elicited a less decrease in BP in most of administration period in the RD group (*P* < 0.05, Figure 3(b)). Notably, the ISO-induced decrease in MAP in RD group restored to its baseline level in second week, but not in Sham, icv Irbe, or icv ENT group (Figure 3(a)).

ISO administration induced significant increase in HR that sustained the whole administration period in both sham and RD rats (*P* < 0.05, Figure 4(a)). The RD, but not icv Irbe and icv ENT, significantly attenuated ISO-induced increase in HR in first 5 days (*P* < 0.05); thereafter, ISO elicited similar increase in HR in all groups (Figure 4(b)).

### 3.4. Effects of Renal Denervation, Central Blockade of AT1-R, or Inhibition of TNF-*α* on ISO-Induced Cardiovascular Variability and Spontaneous Baroreflex

**Figure 5:**
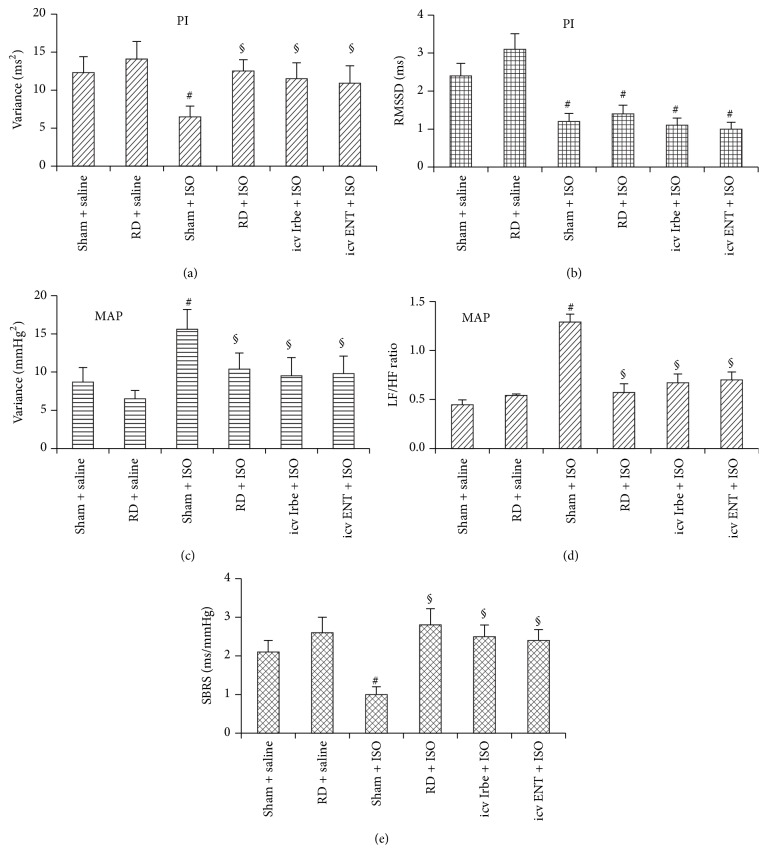
Spectral analysis of pulse interval (PI) variability, mean arterial pressure (MAP) variability, and spontaneous baroreflex sensitivity (SBRS) in rats with sham surgery (Sham), renal denervation (RD), intracerebroventricular (icv) infusion of AT1-R antagonist irbesartan (Irbe), or TNF-*α* inhibitor etanercept (ENT) during saline or isoproterenol (ISO) administration. (a) Variance (heart rate variability); (b) RMSSD, root mean square of successive differences in PI; (c) variance (blood pressure variability); (d) the ratio of low frequency (LF)/high frequency (HF); (e) SBRS (*n* = 6/group; ^#^ *P* < 0.05 versus Sham + saline or RD + saline; ^§^ *P* < 0.05 versus RD + ISO).

Although these parameters did not differ in the 2 groups of rats with saline treatment, overall HR variability (variance of PI) and beat-to-beat variability attributed to parasympathetic modulation (RMSSD of PI) were reduced significantly in ISO treated rats (*P* < 0.05, Figures 5(a) and 5(b)). Conversely, BP variability (variance of MAP) and the LF component (MAP) attributed to sympathetic modulation were markedly increased while the HF component attributed to parasympathetic modulation was decreased in ISO treated rats (*P* < 0.05, Figure 5(c)). As a result, the LF/HF ratio (MAP) was significantly increased (*P* < 0.05, Figure 5(d)). These ISO-induced effects were reversed by RD, icv Irbe, or icv ENT except RMSSD of PI (*P* < 0.05).

SBRS was also markedly reduced in ISO treated rats. This depressed baroreflex sensitivity was prevented by RD, icv Irbe, or icv ENT (*P* < 0.05, Figure 5(e)).

### 3.5. Effects of Renal Denervation, Central Blockade of AT1-R, or Inhibition of TNF-*α* on ISO-Induced Arterial Baroreflex Dysfunction

**Figure 6:**
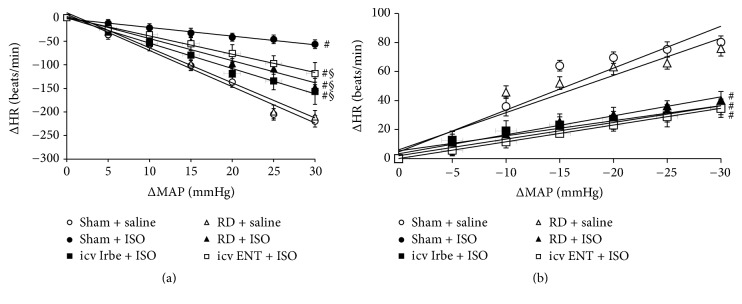
Comparison of decreases in heart rate (HR (a)) to increases in mean arterial pressure (MAP) evoked by phenylephrine (PE) in rats with sham surgery (Sham), renal denervation (RD), intracerebroventricular (icv) infusion of AT1-R antagonist irbesartan (Irbe), or TNF-*α* inhibitor etanercept (ENT) after saline or isoproterenol (ISO) administration. The straight lines were an average representation of the regression lines fit through the data point. (b) Comparison of increases in HR to decreases in MAP evoked by sodium nitroprusside (SNP) (*n* = 6/group; ^#^ *P* < 0.05 versus Sham + saline or RD + saline; ^§^ *P* < 0.05 versus Sham + ISO).

We also evaluated baroreflex function under pharmacological intervention. To observe true differences in baroreflex sensitivity independent of the starting values, the slope values for regression lines relating changes in HR to changes in BP during PE (Figure 6(a)) or SNP (Figure 6(b)) infusion were calculated. The slope values in Sham + saline rats were similar to that in RD + saline rats. ISO administration resulted in a significant decrease in slope values in Sham rats (*P* < 0.05, Table 3). Either RD, icv Irbe, or icv ENT significantly restored the depressed slope value for bradycardia, but not for tachycardia, induced by ISO (*P* < 0.05, Table 3).

### 3.6. Effect of Renal Denervation, Central Blockade of AT1-R, or Inhibition of TNF-*α* on ISO-Induced mRNA Expression of RAS Components and Inflammatory Cytokines in the Brain

**Figure 7:**
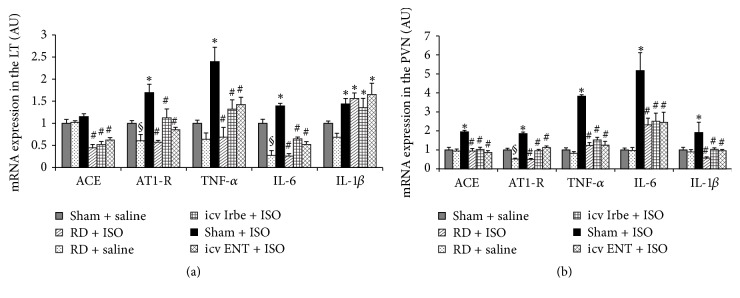
Quantitative comparison of the mRNA expression of renin-angiotensin system components and proinflammatory cytokines in the lamina terminalis (LT (a)) and paraventricular nucleus (PVN (b)) of rats receiving renal denervation (RD), intracerebroventricular (icv) infusion of AT1-R antagonist irbesartan (Irbe), or TNF-*α* inhibitor etanercept (ENT) after isoproterenol (ISO) administration (*n* = 6/group; ^§^ *P* < 0.05 versus Sham + saline; ^*∗*^ *P* < 0.05 versus Sham + saline or RD + saline; ^#^ *P* < 0.05 versus Sham + ISO).

RD itself significantly reduced AT1-R expression in both the LT and PVN and IL-6 in the LT alone (Figures 7(a) and 7(b)).

In LT tissue, ISO resulted in a significant increase in the mRNA expression of the RAS component (AT1-R) and the inflammatory cytokines (i.e., TNF-*α*, IL-6, and IL-1*β*) when compared with saline group (*P* < 0.05, Figure 7(a)). The expression of ACE was not higher after ISO (*P* > 0.05, Figure 7(a)). RD, icv Irbe, or icv ENT significantly inhibited mRNA expression of ACE and attenuated the increased mRNA expression of AT1-R, TNF-*α*, and IL-6 (*P* < 0.05, Figure 7(a)) while the IL-1*β* expression remained higher.

In PVN tissue, ISO also increased the mRNA expression of the RAS components (AT1-R and ACE) and the inflammatory cytokines (TNF-*α*, IL-1*β*, and IL-6) (*P* < 0.05, Figure 7(b)). Either RD, icv Irbe, or icv ENT abolished the increased mRNA expression of these genes (*P* < 0.05, Figure 7(b)).

### 3.7. Effect of Renal Denervation, Central Blockade of AT1-R, or Inhibition of TNF-*α* on ISO-Induced Protein Expressions of ACE and TNF-*α* in the Brain

**Figure 8:**
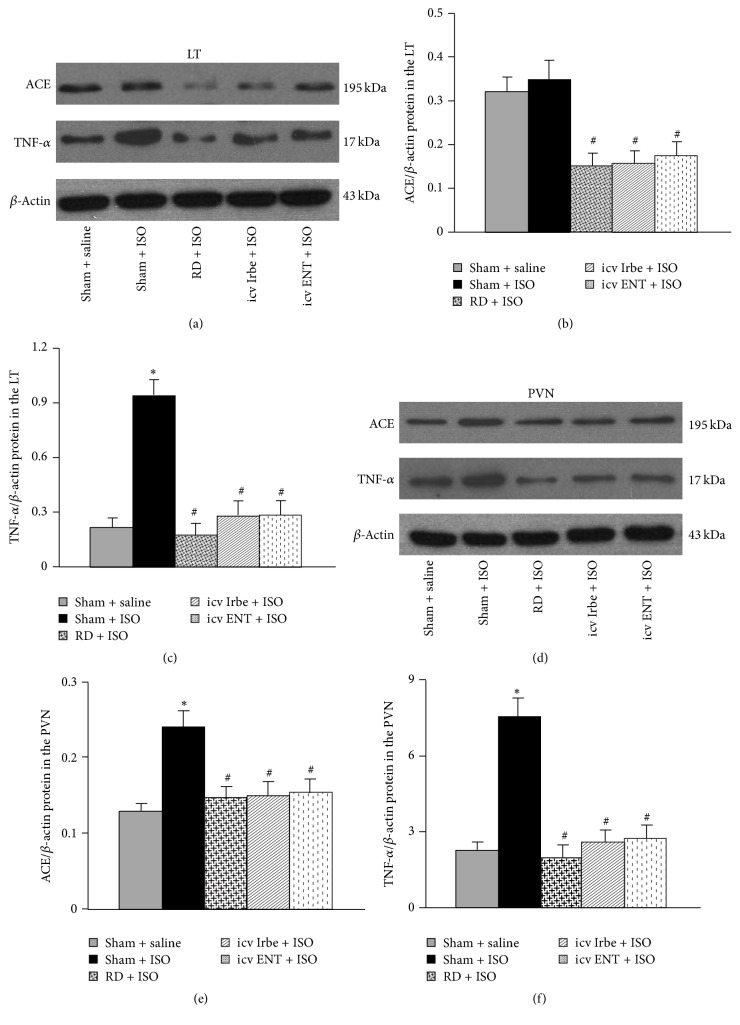
Representative Western blots and quantitative comparison of protein levels for renin-angiotensin system component (ACE) and inflammatory cytokine (TNF-*α*) in the LT ((a)–(c)) and PVN ((d)–(f)) in each group. Values are corrected by *β*-actin and expressed as mean ± SEM (*n* = 5/group; ^*∗*^ *P* < 0.05 versus Sham + saline; ^#^ *P* < 0.05 versus Sham + ISO).

The Western blotting analysis for protein expression confirmed the effects of RD on genomic regulation. ISO treatment resulted in a significant increase in the protein expression of ACE in the PVN, but not in the LT, while TNF-*α* was increased in both the LT and PVN (*P* < 0.05). Either RD, central blockade of AT1-R, or inhibition of TNF-*α* reversed the changes in the protein expression of ACE and TNF-*α* in both the LT and PVN (*P* < 0.05, Figure 8).

## 4. Discussion

Although RD has been shown to improve cardiac function which is accompanied by decreased plasma RAS components and improved autonomic modulation [17, 20]; it is unknown how RD impacts on BP and HR in the development of HF and how interaction between RD and the CNS is involved in the beneficial effects of RD. The major findings of the present study are as follows: (1) RD significantly inhibited the decrease in BP in the development of ISO-induced HF and favorably altered autonomic regulation, including increase in HR variability and BRS and decrease in BP variability; (2) RD significantly inhibited ISO-induced increases in mRNA and protein expression of RAS components and PICs in the LT and PVN, two forebrain nuclei involved in regulation of BP and sympathetic activity; (3) central blockade of either AT1-R or TNF-*α* partially mimicked the beneficial actions of RD in the ISO-induced HF. The results indicate that RD restores BP, autonomic and cardiac dysfunction induced by ISO, and that the central mechanisms involving inhibition of the RAS and PICs contribute to the beneficial effects of RD.

Based on the effectiveness of RD in profound and prolonged reduction of BP and sympathetic outflow as a therapy for drug-resistant hypertension [29, 30], the efficiency of this procedure in the setting of CHF has begun to be assessed extensively. Recent studies have demonstrated that bilateral RD exerts beneficial actions in both animal and patient models of CHF [14, 21], including reduction of myocardial hypertrophy [15], improvement of renal function [31] and cardiac output [32], restoration of autonomic balance [17, 20, 33], and inhibition of RAS activity and inflammation in the myocardium [17, 19].

The capacity of ISO to induce HF has been extensively studied by using different doses and duration of ISO administration [22]. High doses of ISO (150–340 mg/kg in single dose or in two consecutive doses) administration resulted in myocyte necrosis and decreased diastolic and systolic functions [34, 35]. The present study confirmed that continued infusion of a high dose of ISO (40 mg/kg/day for 14 days) considerably worsened both the systolic and the diastolic parameters. Notably, we used telemetric probes to continuously monitor the time course of BP and HR changes during ISO administration although the previous studies have used tail-cuff plethysmography or fluid catheter to measure BP and HR at the end of the experiment, showing a decrease in BP and HR [22, 36, 37]. We found that ISO elicited a sharp decrease in BP in first week and gradually restored in second week but did not return to basal level. In contrast, HR exhibited a significant increase throughout the ISO infusion rather than a decrease shown in previous studies [37]. RD significantly ameliorated cardiac dysfunction and inhibited the decrease in BP and the increase in HR produced by ISO. These favorable effects of RD on BP may be attributed to improvement of cardiac systolic and diastolic function, reduction of total peripheral resistance, and restoration of cardiac output [32, 38]. Our results are consistent with the recent REACH-pilot study showing that, in patients with CHF, bilateral RD increases exercise tolerance and provides patients with symptomatic improvement, whereas BP remains stable [16].

Using spectral analysis, the measurement of variability in the time and frequency domain allows identification of sequence of successive PI or MAP values. These rhythmic oscillations at various frequencies reflect sympathetic or parasympathetic modulation on the cardiovascular system in physiological and pathophysiological states [24, 25]. In the present study, the ISO treated animals had decreased HR variability and RMSSD and increased LF/HF ratio and BP variability, suggesting a higher sympathetic and a lower parasympathetic modulation in heart and blood vessels. BRS can be assessed by analyzing the spontaneous changes in MAP and PI using the sequence method [39] or by measuring ratio of bradycardia response to rise in BP and ratio of tachycardia response to fall in BP using pharmacological intervention [26]. Previous studies have shown that baroreflex is impaired in animals with ISO-induced or pacing-induced HF [20, 40]. Using both analyses of sequence method and pharmacological intervention, our study confirmed and extended these previous studies by showing that there was a blunted BRS after ISO administration.

Autonomic function and baroreflexes normally contribute to cardiovascular homeostasis by maintaining BP. Conversely, blunted arterial baroreflex regulation, decreased HR variability, and increased BP variability have been directly implicated as enhanced risk factors for sudden cardiac death and cardiovascular mortality [41]. Therefore, it can be speculated that, in the present study, ISO-induced autonomic unbalance and baroreflex dysfunction may contribute to sustainedly decreased BP and increased HR during ISO administration. RD favorably modulated most of autonomic parameters studied, suggesting that RD restorations of autonomic balance and BRS are involved in maintenance of stable BP and in improvement of cardiac function during ISO-induced HF. However, it should be noted that RD had no effects on ISO-induced decreases in RMSSD and in tachycardic response to decrease in BP with SNP. Due to these two measures reflecting parasympathetic-mediated modulation of HR, these results suggest that RD inhibition of sympathetic activity plays a dominant role in restoring autonomic balance.

It has been shown that the PVN is an important neuroendocrine and preautonomic output nucleus inside blood-brain barrier (BBB) that integrates and responds to a variety of neural and humoral signals regulating sympathetic drive and extracellular fluid volume [42]. The LT including the SFO and OVLT, sensory circumventricular organs lacking the normal BBB, has also been documented as a CNS structure involved in cardiovascular regulation [5]. The SFO and OVLT communicate with the PVN through efferent projections to this hypothalamic nucleus. This descending neural pathway provides a means through which information about systemic cardiovascular factors such as the RAS and PICs may reach hypothalamic neurons residing inside the BBB [43]. It has been shown that HF is associated with exaggerated sympathetic activity which is due to an imbalance between inhibitory and excitatory mechanisms within specific areas in the CNS such as the LT and PVN [44]. Elevated levels of RAS components and PICs and synergistic interaction between the RAS and PICs in these forebrain nuclei play a pivotal role in central processing of sympathetic nerve activity during HF [2, 8]. Previous studies have demonstrated that ISO-induced HF is associated with increased levels of RAS components and PICs in peripheral circulating system [2, 34, 35]. In the present study, we found that ISO administration significantly increased the mRNA and protein expressions of the RAS and PICs in the LT and PVN, which was remarkably attenuated by RD. Central blockade of either the RAS or inflammation mimicked not only the beneficial effects of RD on ISO-induced autonomic and cardiac dysfunctions, but also the inhibitory effects on ISO-elicited increases in mRNA and protein expression of the RAS and PICs in the LT and PVN. These results provide insights into the key mechanisms by which RD restores autonomic balance and cardiac function through its capacity to inhibit ISO-induced activation of the RAS and PICs in the cardiovascular neural network.

There are some potential limitations to this study. Our study determined the mRNA and protein expressions in forebrain cardiovascular nuclei such as the LT and PVN after RD and ISO treatment. However, the protective role of RD in the development of ISO-induced HF cannot be attributed solely to the changes in gene expression in these two forebrain nuclei. The brainstem nuclei involved in regulation of baroreflex and autonomic function such as the nucleus of solitary tract (NTS) and RVLM [45] may also contribute to the protective role of RD in ISO-induced HF. In addition, there is a prompt and direct interaction of renal afferent input with baroreceptor and cardiac chemoreceptors at the level of the PVN to dictate overall sympathetic tone [46]. RD procedure involves denervation of both the renal efferent sympathetic nerves and afferent sensory nerves. Thus, the studies on identification of the protective action of efferent sympathetic nerves versus afferent sensory nerves should be performed in the future.

**Figure 9:**
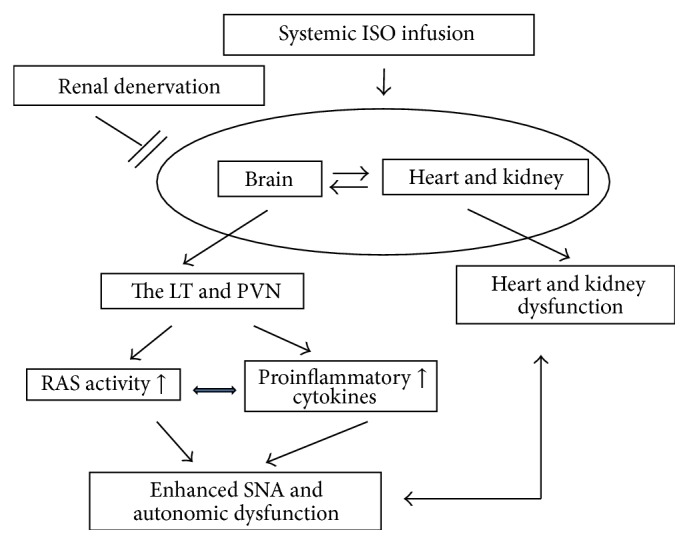
Schematic diagram showing possible mechanisms by which renal denervation (RD) might ameliorate isoproterenol- (ISO-) induced heart failure. LT, the lamina terminalis; PVN, the paraventricular nucleus of hypothalamus; SNA, sympathetic nerve activity.

Taken together, the present study demonstrated that RD inhibits ISO-induced increases in mRNA and protein expression of the RAS components and PICs in the brain and that this is associated with the improved cardiac and autonomic function, as illustrated in Figure 9. The results indicate that RD-elicited beneficial effects involve the interaction between RD and the CNS in the ISO-induced HF. The present study provides new evidence supporting that RD is a promising therapeutic strategy for HF with maintenance of a stable BP.

## Figures and Tables

**Table 1:** Primer sequences for real-time PCR.

Gene	Forward primer	Reverse primer	Product size (bp)

GAPDH	TGACTCTACCCACGGCAAGTTCAA	ACGACATACTCAGCACCAGCATCA	141
ACE	GTGTTGTGGAACGAATACGC	CCTTCTTTATGATCCGCTTGA	187
AT1-R	CTCAAGCCTGTCTACGAAAATGAG	GTGAATGGTCCTTTGGTCGT	188
TNF-*α*	GCCGATTTGCCACTTCATAC	AAGTAGACCTGCCCGGACTC	209
IL-1*β*	AGCAACGACAAAATCCCT GT	GAAGACAAACCGCTTTTCCA	209
IL-6	GCCTATTGAAAATCTGCTCTGG	GGAAGTTGGGGTAGGAAGGA	160

ACE, angiotensin-converting enzyme; AT1-R, angiotensin II type 1 receptor; IL-6, interleukin-6; IL-1*β*, interleukin-1*β*; TNF-*α*, tumor necrosis factor-*α*.

**Table 2:** Changes in cardiac parameters in control and heart failure rats with renal denervation (RD) and intracerebroventricular (icv) infusion of AT1-R antagonist irbesartan (Irbe) or of TNF-*α* inhibitor etanercept (ENT).

	Control (*n* = 6/group)		Heart failure (*n* = 6/group)			
	Sham + saline	RD + saline	Sham + ISO	RD + ISO	icv Irbe + ISO	icv ENT + ISO
HW/BW (g/kg)	3.65 ± 0.09	3.81 ± 0.07	5.26 ± 0.05^*∗*^	4.01 ± 0.08^#^	4.21 ± 0.10^#^	4.31 ± 0.15^#^
HR (beats/min)	361.2 ± 12.3	359.6 ± 10.0	460.9 ± 11.5^*∗*^	452.7 ± 8.3^*∗*^	465.3 ± 12.4^*∗*^	458.9 ± 9.8^*∗*^
LVSP (mmHg)	139.6 ± 2.6	140.9 ± 4.1	107.5 ± 4.7^*∗*^	126.5 ± 5.2^*∗*#^	128.6 ± 4.5^*∗*#^	127.5 ± 3.5^*∗*#^
LVEDP (mmHg)	3.9 ± 0.4	3.1 ± 0.3	15.6 ± 2.5^*∗*^	8.8 ± 1.9^*∗*#^	9.5 ± 2.1^*∗*#^	10.2 ± 1.5^*∗*#^
+dp/dt_max_ (mmHg/s)	5842 ± 278	5722 ± 203	3201 ± 289^*∗*^	4351 ± 397^*∗*#^	4024 ± 265^*∗*#^	4265 ± 352^*∗*#^
−dp/dt_min_ (mmHg/s)	−4335 ± 146	−4375 ± 275	−2388 ± 289^*∗*^	−3402 ± 283^*∗*#^	−3254 ± 216^*∗*#^	−3198 ± 189^*∗*#^

ISO, isoproterenol; HW/BW, heart weight to body weight ratio; HR, heart rate; LVSP, left ventricular systolic pressure; LVEDP, left ventricular end-diastolic pressure; +dP/dt_max_, maximal rate of rise of LVP; −dP/dt_min_, minimal rate of decrease of LVP. ^*∗*^ *P* < 0.05 versus Sham or RD + saline; ^#^ *P* < 0.05 versus Sham + ISO.

**Table 3:** The slopes of baroreflex function in control and heart failure rats with renal denervation (RD) and intracerebroventricular (icv) infusion of AT1-R antagonist irbesartan (Irbe) or of TNF-*α* inhibitor etanercept (ENT).

	Baroreflex slopes (*n* = 6/group)					

	Sham + saline	RD + saline	Sham + ISO	RD + ISO	icv Irbe + ISO	icv ENT + ISO
PE	−8.15 ± 1.21	−7.46 ± 1.63	−1.85 ± 0.67^#^	−4.69 ± 1.06^#§^	−5.38 ± 0.84^#§^	−3.87 ± 0.71^#§^
SNP	2.88 ± 0.63	2.56 ± 0.44	1.15 ± 0.25^#^	1.31 ± 0.22^#^	1.04 ± 0.13^#^	1.15 ± 0.26^#^

ISO, isoproterenol; PE, phenylephrine; SNP, sodium nitroprusside. ^#^ *P* < 0.05 versus Sham or RD + saline; ^§^ *P* < 0.05 versus Sham + ISO.
